# *Clostridioides difficile* infection is associated with differences in transcriptionally active microbial communities

**DOI:** 10.3389/fmicb.2024.1398018

**Published:** 2024-04-12

**Authors:** Jeremy R. Chen See, Jillian Leister, Justin R. Wright, Peter I. Kruse, Mohini V. Khedekar, Catharine E. Besch, Carol A. Kumamoto, Gregory R. Madden, David B. Stewart, Regina Lamendella

**Affiliations:** ^1^Juniata College, Huntingdon, PA, United States; ^2^Wright Labs LLC, Huntingdon, PA, United States; ^3^Molecular Biology and Microbiology, Tufts University, Boston, MA, United States; ^4^University of Virginia School of Medicine, Charlottesville, VA, United States; ^5^Department of Surgery, Southern Illinois University School of Medicine, Springfield, IL, United States

**Keywords:** *Clostridioides difficile*, metatranscriptomics, fungi, human microbiome, mycobiome

## Abstract

*Clostridioides difficile* infection (CDI) is responsible for around 300,000 hospitalizations yearly in the United States, with the associated monetary cost being billions of dollars. Gut microbiome dysbiosis is known to be important to CDI. To the best of our knowledge, metatranscriptomics (MT) has only been used to characterize gut microbiome composition and function in one prior study involving CDI patients. Therefore, we utilized MT to investigate differences in active community diversity and composition between CDI+ (*n* = 20) and CDI− (*n* = 19) samples with respect to microbial taxa and expressed genes. No significant (Kruskal-Wallis, *p* > 0.05) differences were detected for richness or evenness based on CDI status. However, clustering based on CDI status was significant for both active microbial taxa and expressed genes datasets (PERMANOVA, *p* ≤ 0.05). Furthermore, differential feature analysis revealed greater expression of the opportunistic pathogens *Enterocloster bolteae* and *Ruminococcus gnavus* in CDI+ compared to CDI− samples. When only fungal sequences were considered, the family Saccharomycetaceae expressed more genes in CDI−, while 31 other fungal taxa were identified as significantly (Kruskal-Wallis *p* ≤ 0.05, log(LDA) ≥ 2) associated with CDI+. We also detected a variety of genes and pathways that differed significantly (Kruskal-Wallis *p* ≤ 0.05, log(LDA) ≥ 2) based on CDI status. Notably, differential genes associated with biofilm formation were expressed by *C. difficile*. This provides evidence of another possible contributor to *C. difficile*’s resistance to antibiotics and frequent recurrence *in vivo*. Furthermore, the greater number of CDI+ associated fungal taxa constitute additional evidence that the mycobiome is important to CDI pathogenesis. Future work will focus on establishing if *C. difficile* is actively producing biofilms during infection and if any specific fungal taxa are particularly influential in CDI.

## 1 Introduction

Hospital-acquired *Clostridioides difficile* infection (CDI) accounts for approximately 300,000 hospitalizations per year (Bobo et al., 2011) with mortality rates as high as 16.7% during outbreaks (Dubberke et al., 2008), and it has surpassed methicillin-resistant *Staphylococcus aureus* as the highest incidence hospital-acquired infection in the United States (Lessa et al., 2015), all resulting in billions of dollars in annual healthcare costs (Dubberke and Olsen, 2012). Antibiotics remain the most important risk factor for CDI due to their mechanism of action lacking species-level specificity, with their broad impact on gut ecology creating an intestinal dysbiosis in which *C. difficile* has a selective advantage for population growth (Aslam et al., 2005; Shivashankar et al., 2014). Antibiotics are also the most common intervention for CDI, with unacceptably high recurrence rates of 15%–30% after first treatment, representing another manifestation of their causal influence in creating gut microbial environments that promote this disease state (Choi et al., 2011). These observations underscore the importance of gut dysbiosis in the pathogenesis of CDI, which has implications both for disease prevention and disease treatment. A more precise understanding of the dysbiosis of CDI may, therefore, impact the prevention and treatment of this disease.

The majority of microbiome studies on CDI have used amplicons (16S rRNA and/or ITS) to characterize the dominant microbial communities in CDI (Antharam et al., 2013; Sangster et al., 2016; Lamendella et al., 2018; Zuo et al., 2018). However, the studies that relied solely on 16S rRNA data were unable to comment on the possible role of fungi in the pathogenesis of CDI. More recent investigations, including several by our team, suggest that fungi may have a role in CDI (Lamendella et al., 2018; Markey et al., 2018; Stewart et al., 2019). These data suggest that pre-colonization of mice with *C. albicans* lowers their susceptibility to CDI (Markey et al., 2018). Several human cohort studies also demonstrate the consistent presence of fungi, especially *C. glabrata*, in patients with CDI, while CDI− patients with diarrhea and comparable antibiotic exposures lack this fungal enrichment (Lamendella et al., 2018; Stewart et al., 2019). Furthermore, while predictive functional tools exist, namely PICRUSt2 (Douglas et al., 2020) and Tax4Fun2 (Wemheuer et al., 2020), 16S rRNA and ITS data do not allow for the direct detection of expressed genes, and neither of those tools provide any information on which of the predictive functions were likely being expressed.

Shotgun metagenomics can directly detect genes, but it does not distinguish between genes that were expressed vs. those that were only present. In contrast, metatranscriptomics (MT) both overcomes limitations associated with amplicon sequencing and allows us to focus on transcriptionally active microbes, as well as the genes they were expressing. Therefore, we once more utilized MT sequencing to characterize microbial communities within the context of CDI. In this study, we sought to examine potential differences in overall community diversity and identify differentially active microbes and differentially expressed functions based on CDI status. Additionally, building on previous work, we were able to link specific genes and pathways to fungal expression. Despite only having information on CDI status, we were able to detect multiple differences in active community composition and function, which seem to have remained consistent regardless of possible confounding variables.

## 2 Materials and methods

### 2.1 Sample collection

The Institutional Review Board at the University of Virginia approved this study (IRB-HSR# 21646) with waiver of consent, as samples were de-identified remnants. Diarrheal samples from CDI+ (*n* = 20) and CDI− (*n* = 20) patients collected by the University of Virginia Medical Center between September 2019 and August 2020. CDI was diagnosed based on the presence of a conserved sequence of the *tcdB* gene, as determined by Xpert® *C. difficile* assay (Cepheid, Sunnyvale, CA, United States). To be included in the study, patients had to be at least 18 years old. Samples were stored in a −80°C freezer after collection until further processing.

### 2.2 RNA extraction, library preparation, and sequencing

RNA was extracted from samples using the ZymoBIOMICS RNA Miniprep Kit (Zymo Research, Irvine, CA, United States) according to the manufacturer’s protocol with the following exceptions: 1 volume of lysis buffer was used, the optional DNase I treatment was completed, and extracts were eluted with 50 uL of DNase/RNase free water. After extraction, quantification was conducted using an Invitrogen Qubit 4 Fluorometer and Qubit RNA High Sensitivity Assay Kit (ThermoFisher Scientific, Waltham, MA, United States).

RNA was then processed to make MT libraries using the NEBNext Ultra II RNA Library Prep Kit for use with Illumina (New England Biolabs, Ipswich, MA, United States), which utilizes a random priming approach for reverse transcription. The protocol specifically for use with purified mRNA was followed. Depletion of rRNA was not performed.

Libraries were quality checked using an Agilent 2100 Bioanalyzer and DNA High Sensitivity kit. Results from these steps were used to pool samples in an equimolar ratio. The pool was then gel purified using a 2% agarose gel and the Qiagen QIAquick gel extraction kit (Qiagen, Germantown, MD, United States). Following purification, the pool was sequenced using an Illumina NextSeq 550 platform to produce 2 × 150 bp reads by Wright Labs LLC (Huntingdon, PA, United States).

### 2.3 Bioinformatics and statistical analyses

#### 2.3.1 Quality checking and sequence annotation

Quality was checked in the raw sequence data using VSEARCH (Rognes et al., 2016). Following initial quality evaluation, fastp (Chen et al., 2018) was used to filter the data with a sliding window of 4 with a minimum average Phred Q score of 20 in which a window not meeting the average would result in the window as well as the remainder of the sequence being dropped. Sequences shorter than 90 bp after filtering were discarded.

Kraken2 (Wood et al., 2019) was subsequently used to taxonomically annotate the remaining sequences with a database, including its standard libraries, as well as fungi. Species-level annotations were then collated into a table for use with downstream analyses. Counts for the species *Homo sapiens* were excluded to avoid human contamination impacting results. Another table was created by subsetting the full species table to only include fungal species.

The Kraken2-annotated sequences, except for those identified as *Homo sapiens*, were paired with PEAR (Zhang et al., 2014) and dereplicated with VSEARCH. Emapper v2.0 (Huerta-Cepas et al., 2017) using the eggNOG 5.0 database (Huerta-Cepas et al., 2019) was run on the dereplicated sequences. Hits against KEGG Orthologs (KOs) were used to create a table of reads per kilobase (rpk) values for downstream analysis. During table creation, the counts were rpk normalized by dividing sequence occurrences by three times the respective length of the protein they were annotated as (to convert from amino acid length to nucleotide length) and multiplying the quotient by 1,000. The full KOs table was subsetted to create another table containing only annotations of fungal sequences.

Additionally, a fifth dataset containing predicted metabolites based on the paired filtered data was created for use with LEfSe analysis (Segata et al., 2011). The filtered paired sequences were used with HUMAnN3 (Beghini et al., 2021) to generate UniRef90 annotations as input for MelonnPan (Mallick et al., 2019) predicted metabolite analysis using their pre-trained model (Franzosa et al., 2019). The “Unmapped” row was removed from the table, and UniRef90 counts were converted to relative abundances prior to MelonnPan analysis.

In total, five datasets were generated and used for all subsequent analyses: total active species, fungal active species, total expressed genes, fungal expressed genes, and predicted metabolites. Of the five, total active species and total expressed genes were used with all subsequent analyses, while the other three were only used for differential feature analysis. One sample (73) was omitted from all analyses due to yielding fewer than 500,000 raw sequences.

#### 2.3.2 Alpha diversity analysis

Alpha diversity was calculated by subsampling the active microbial taxa and expressed genes dataset tables at 10 different depths, ranging from 99,000 to 990,000 for the active microbial species dataset (Supplementary Figure S1) and 590 to 5,900 for the expressed genes dataset (Supplementary Figure S2). A single sample (68) was omitted from alpha diversity analyses with the microbial taxa dataset to facilitate a higher rarefaction depth as opposed to having to use a maximum depth of 117,600 instead to retain it. Twenty iterations were performed at each depth to obtain average alpha diversity values for the different metrics [Observed Features and Pielou’s Evenness (Pielou, 1966)]. Averages for the greatest depth were used to see if any alpha diversity metrics differed significantly based on CDI status (Kruskal-Wallis, *p* ≤ 0.05) with QIIME2 (Bolyen et al., 2019).

#### 2.3.3 Beta diversity analysis

Beta diversity analyses were conducted after the tables had first undergone counts per million normalization to mitigate differences between samples based on sequencing depth. The Bray-Curtis distance metric (Sørensen, 1948) was used to create a distance matrix for both datasets. The resulting distance matrices were visualized as Principal Coordinates Analysis plots with 95% confidence intervals around the centroids using the ggordiplot package (Quensen, 2020) through R (R Core Team, 2023). Statistical differences between sample groupings based on CDI status were evaluated as well (PERMANOVA, *p* ≤ 0.05) through QIIME2.

#### 2.3.4 Differential feature analysis

Differential feature analysis was performed using LEfSe (Segata et al., 2011) to identify features (genes, pathways, predicted metabolites, and taxa) that had significantly different abundances based on CDI status. For all datasets, the table was normalized with the counts per million (CPM) method. Only features identified as having significantly differential abundance (Kruskal-Wallis, *p* ≤ 0.05) with a log(LDA) score of at least 2.0 were considered to be enriched, with the exception of the predicted metabolites dataset in which features only had to differ significantly based on Kruskal-Wallis. Levene’s test was performed using the rstatix package (Kassambara, 2020) in R with CPM-normalized values for differential features identified within the active microbial species dataset, as several of the most differential taxa seemed to be highly variable.

SparseDossa2 (Ma et al., 2021) was used to simulate 1,000 tables with various minimum fold change differences for the active microbial taxa and expressed genes datasets in order to assess the corresponding change required to achieve 80% power for LEfSe to detect significant features. Each simulated table consisted of 39 samples, split into two groups of sizes 19 and 20, and was based on a SparseDossa2 model fitted for the respective data type.

For every simulation, the active microbial taxa simulated tables used 17 of the 346 species (5%) that were present in at least 30% of the samples with a minimum average relative abundance of 0.01% to model differential features and 76 of the 1,515 KOs (5%) meeting those requirements were used for each simulation based on the expressed genes dataset. Simulated samples had an average depth of 10,000,000 for both the active microbial taxa and expressed genes datasets. All simulated tables were CPM-normalized prior to being used as input for LEfSe and subject to the same criteria as the observed data to be considered differential. Differential features associated with the expected group were counted as true positives.

#### 2.3.5 Differential contributors analysis

The stratified gene contribution table was subsetted to include only genes of interest. The rpk-normalized values were then converted to relative abundances such that for each sample, the values of the taxa contributing to the gene(s) of interest summed to 100. Wilcoxon rank sum tests were then used to assess the significance of differences in relative contribution based on CDI status.

#### 2.3.6 Machine learning

Random forest models were created to assess how well the features identified as differential by LEfSe could be used to predict CDI status compared to models trained on the full datasets. The models were generated with Scikit-learn in Python based on 500 decision trees (Pedregosa et al., 2011). They were evaluated using k-fold cross-validation with five repetitions of 10 folds. The datasets used for model creation include the full species datasets and the species identified as differential by LEfSe. Those two datasets were used to generate additional models after excluding *Clostridioides difficile*. Models were also generated using the full genes dataset and the genes identified as differential. All datasets were subject to CPM normalization prior to model creation. Feature importance was evaluated using Gini importance, and the 10 most important features were plotted for all models.

#### 2.3.7 CoNet analysis

Cooccurrence networks for the active microbial taxa datasets were generated with CoNet (Faust and Raes, 2016) in Cytoscape (Shannon et al., 2003), using automatic thresholds for Spearman correlation (Spearman Rank Correlation Coefficient, 2008), Bray-Curtis (Sørensen, 1948), and Kullback–Leibler (Kullback and Leibler, 1951), with an edge selection parameter of 100 and a minimum occurrence of 10 samples for a feature to be considered. The *p*-values from the individual methods were combined with the Brown method (Brown, 1975), and Benjamini-Hochberg (Benjamini and Hochberg, 1995) p-value correction was performed with a cutoff of 0.05 for significance. Networks were created for CDI+ Total Active Species and CDI− Total Active Species datasets and then juxtaposed using CytoGEDEVO (Malek et al., 2016).

## 3 Results

### 3.1 Description of sequencing results

**Figure 1:**
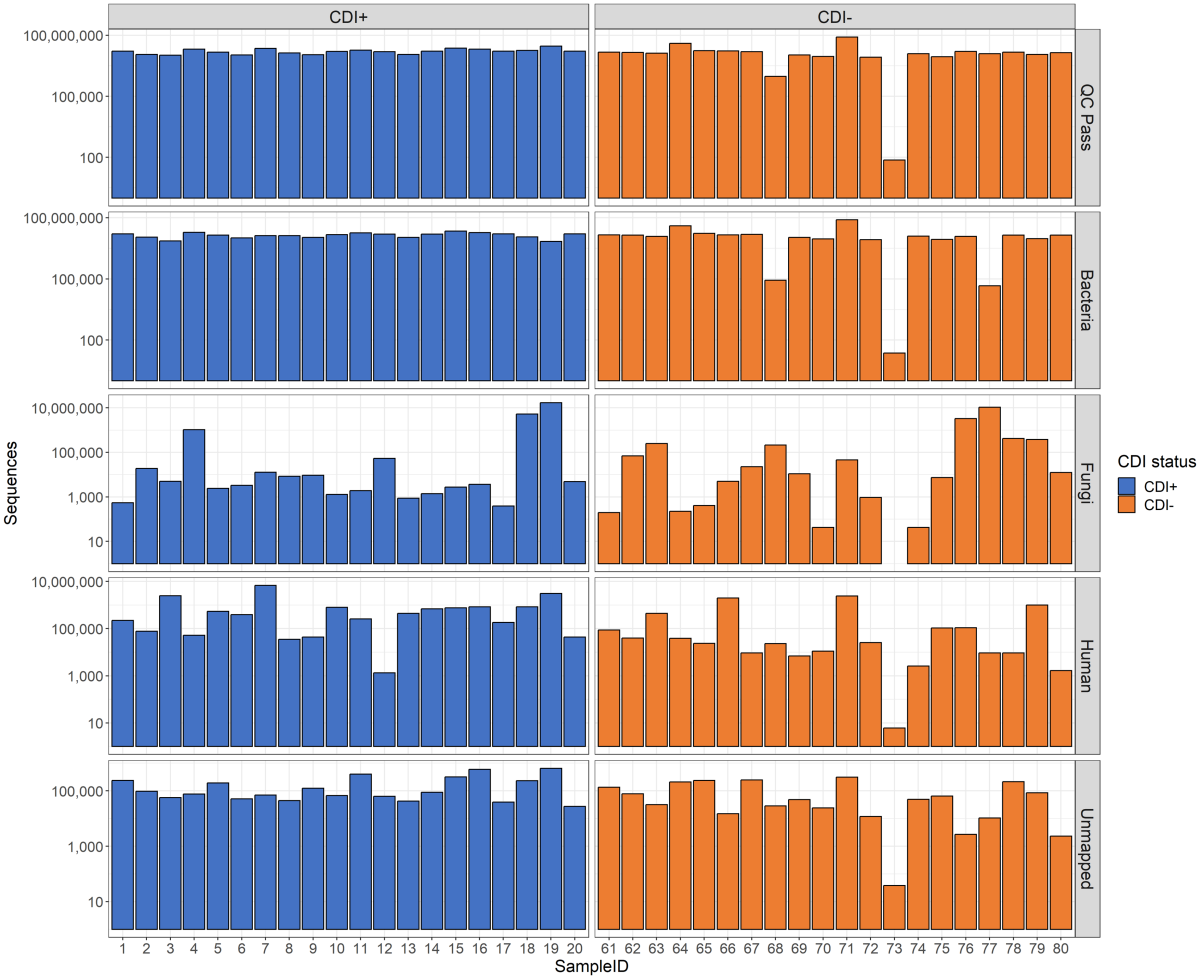
Bar plots of sequencing results. The number of sequences retained after quality control (QC Pass), annotated as Bacteria (Bacteria), annotated as Fungi (Fungi), annotated as *Homo sapiens* (Human), and unidentified by Kraken2 (Unmapped) are shown. Raw sequence counts are shown on the y-axis. The x-axis shows individual samples. Each patient was associated with a single sample.

In total, 40 samples were subjected to MT sequencing, yielding 759,555,378 raw sequences, and after quality filtration, 639,700,992 sequences remained. Of those samples, 39 (CDI + =20, CDI− = 19) had enough data for downstream analysis after quality filtering (range 942,194–82,265,454 sequences per sample, Figure 1).

### 3.2 Richness and evenness

**Figure 2:**
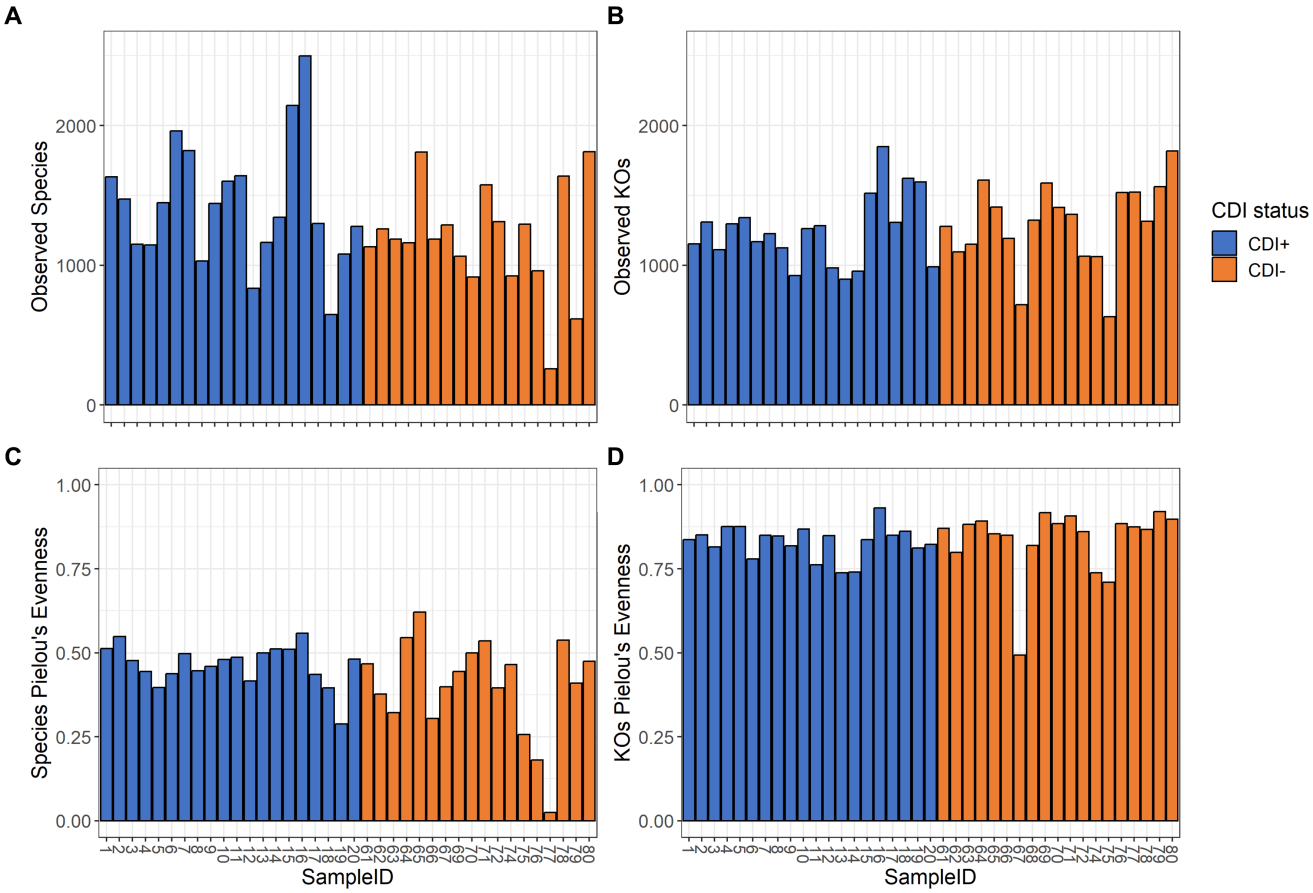
Alpha diversity values for the active composition and expressed genes datasets based on the Observed Features and Pielou’s Evenness metrics per sample. Samples are colored based on CDI status. Sample 68 was omitted from the active microbial species analyses to allow for a greater maximum rarefaction depth. **(A)** Observed Features within the active microbial species dataset. **(B)** Observed Features within the expressed genes dataset. **(C)** Pielou’s evenness within the active microbial species dataset. **(D)** Pielou’s evenness within the expressed genes dataset. Alpha diversity did not differ significantly (Kruskal-Wallis, *p* ≤ 0.05) based on CDI status within either dataset by either metric.

Pielou’s evenness values within the expressed genes dataset tended to be higher in CDI− samples compared to the CDI+ cohort, though the difference was not significant (Table 1). Overall, alpha diversity was variable among samples but did not differ significantly between CDI+ and CDI− for the active microbial taxa or expressed genes datasets based on either the Pielou’s evenness or Observed Features metrics (Figure 2).

**Table 1:** Alpha diversity results for the Observed Features and Pielou’s evenness metrics.

Metric	Dataset	CDI− mean	CDI+ mean	H	Kruskal-Wallis *p*-value
Observed features	Total active species	1188.278 (±91.799)	1431.865 (±98.996)	2.585	0.108
Observed features	Total expressed genes	1297.142 (±68.824)	1245.733 (±56.513)	0.967	0.325
Pielou’s evenness	Total active species	0.403 (±0.034)	0.464 (±0.014)	1.969	0.161
Pielou’s evenness	Total expressed genes	0.838 (±0.023)	0.831 (±0.011)	3.651	0.056

Significance was evaluated using Kruskal-Wallis tests through QIIME2. Mean values with standard error are reported.

### 3.3 Differential active microbial communities

**Figure 3:**
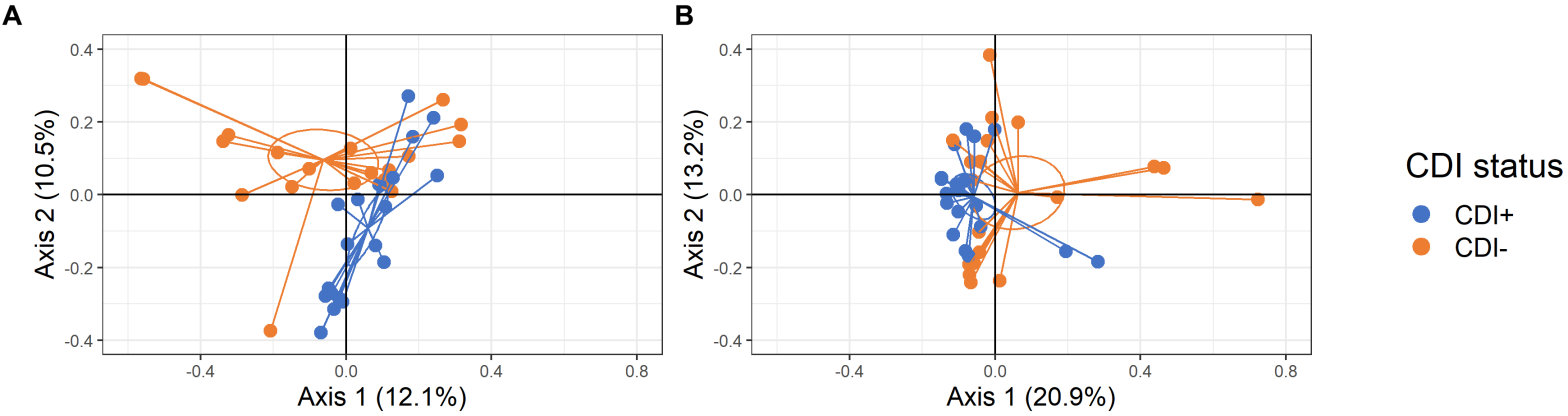
PCoA plots of samples colored by CDI status with centroids shown within 95% confidence interval ellipses based on Bray-Curtis distances calculated with active microbial species **(A)** and expressed genes **(B)**.

Significant clustering based on CDI status was observed for both active taxa and expressed gene datasets (PERMANOVA, *p* = 0.005 and *p* = 0.013, respectively; Figure 3). Subsequent LEfSe analysis identified the microbial taxa and KEGG Orthologs (KOs) driving differential clustering.

**Figure 4:**
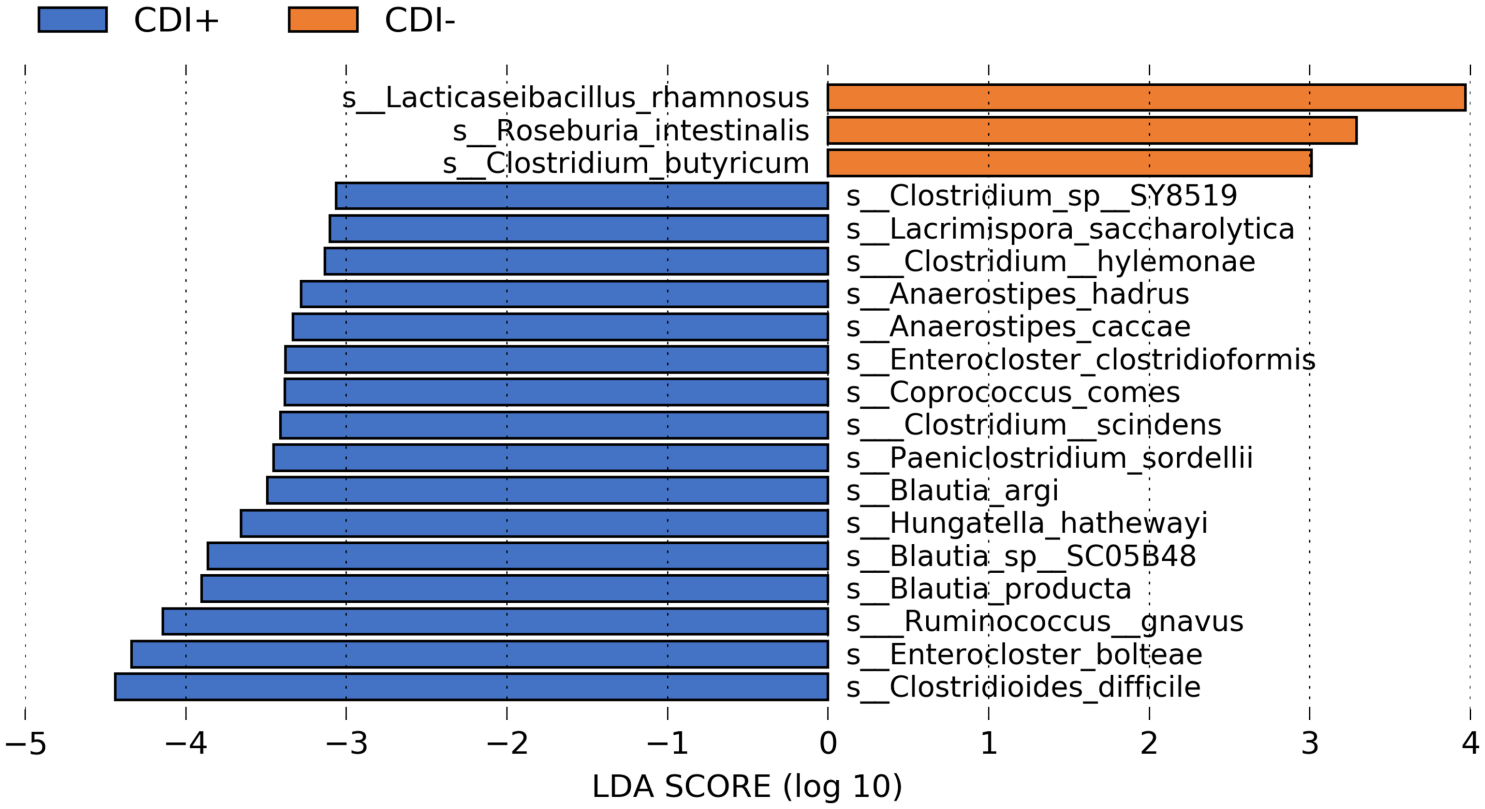
Bar plot of LEfSe results showing highly differential active species (LDA ≥ 3.0, Kruskal-Wallis *p* ≤ 0.05) based on CDI status. See Supplementary Table S1 for all active taxa identified as differential by LEfSe.

The commensal bacteria *Clostridium butyricum*, *Lacticaseibacillus rhamnosus*, and *Roseburia intestinalis* were the most differentially active microbial species in CDI− (Figure 4). However, variability in the expression of all three taxa within CDI− was relatively high (Supplementary Table S1). In contrast, though its corresponding LDA score was lower, *Lactiplantibacillus plantarum* was more consistently active in the CDI− samples (Supplementary Table S1).

Multiple butyrate producers were identified as being more active in CDI+, including *Anaerostipes hadrus* (Allen-Vercoe et al., 2012), *Coproccocus comes* (Louis and Flint, 2009), and *Roseburia hominis* (Louis and Flint, 2009), and all three were found to express genes within the Butanoate Metabolism pathway (ko00650) in this study. However, the taxa *Clostridioides difficile*, *Enterocloster bolteae*, and *Ruminococcus gnavus* were the three most differentially active species in CDI+ (Kruskal-Wallis, *p* ≤ 0.05, log(LDA) ≥ 3.0), with *C. difficile* being the most differential (Supplementary Figure S3). *Clostridium scindens* was also identified as being more active in CDI+.

**Figure 5:**
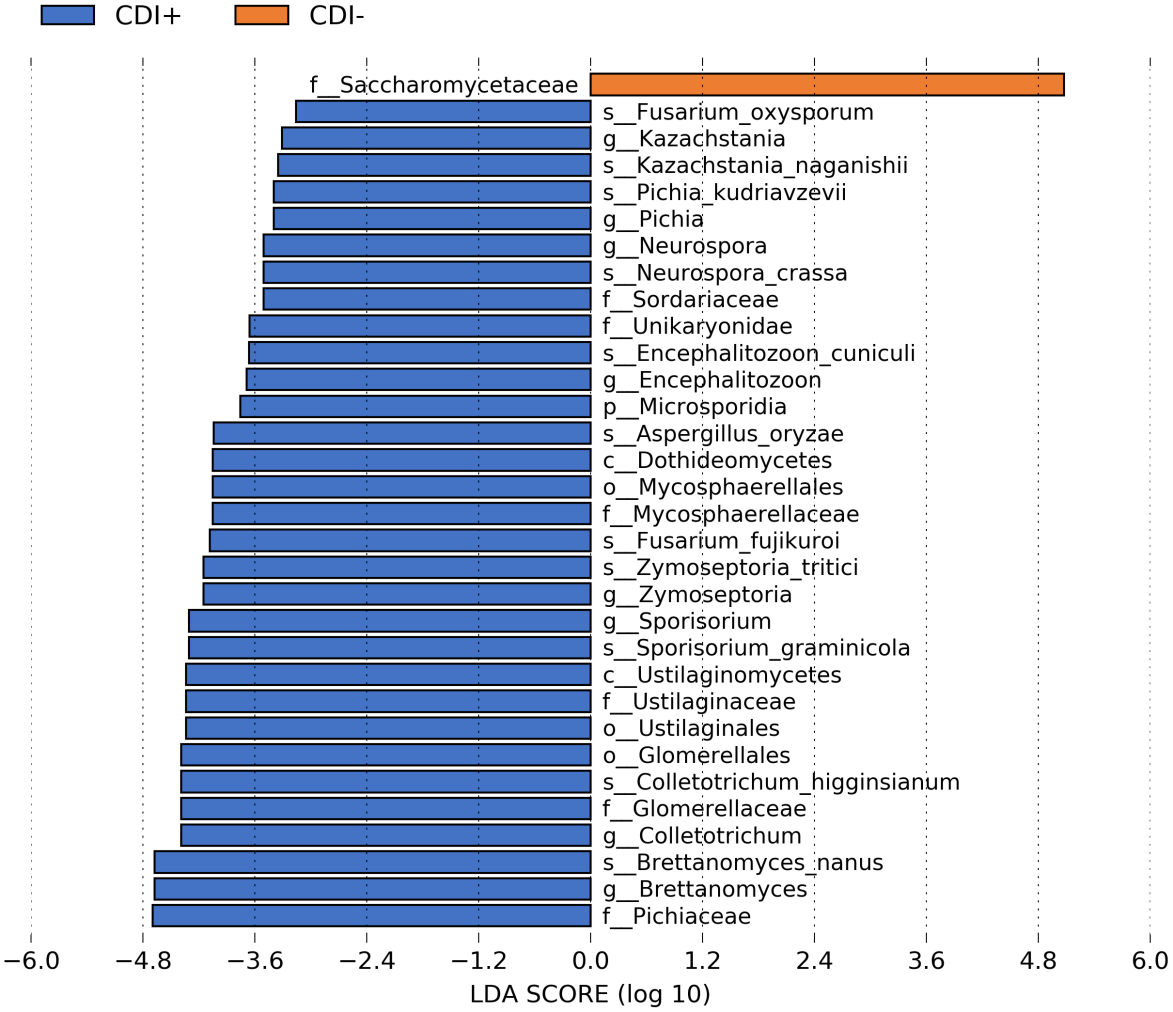
Bar plot of LEfSe results showing differential active fungal taxa (LDA ≥ 2.0, Kruskal-Wallis *p* ≤ 0.05) based on CDI status.

When our microbial taxa table was subsetted to include only fungal taxa, the family Saccharomycetaceae was more active in CDI− samples (Figure 5). In contrast, 31 fungal taxa were more active in CDI+ (Supplementary Table S2). Several differential genes (Supplementary Table S3) and pathways (Supplementary Table S4) were also identified within the fungi functional gene datasets. However, we recovered relatively low numbers of fungal sequences (Supplementary Figure S4).

More differential genes were identified within the CDI+ cohort in the total expressed genes dataset (CDI+ genes = 99, CDI− genes = 31), including several that were especially of interest due to their involvement with biofilm formation or sporulation and their expression by *C. difficile* (Table 2). However, a greater number of pathways were expressed associated as differential in CDI− (Supplementary Table S5). Interestingly, 22 of the genes more expressed in CDI+ code for ribosomal subunits (Supplementary Table S6). The most significantly different contributors to those genes were taxa significantly more active in CDI+ (Supplementary Table S7).

**Table 2:** Differential CDI+ KEGG Orthologs (KOs) of interest due to their potential to facilitate CDI recurrence.

KO	Reason	Total Samples	Samples associated with *C. difficile* expression
K03073: preprotein translocase subunit SecE	Biofilm formation	38	13
K03075: preprotein translocase subunit SecG	Biofilm formation	36	15
K03666: host factor-I protein	Biofilm formation	28	7
K06334: spore coat protein JC	Sporulation	29	10
K06412: stage V sporulation protein G	Sporulation	27	14
K06418: small acid-soluble spore protein A (major alpha-type SASP)	Sporulation	29	21

The KOs listed here were all expressed by *Clostridioides difficile* in at least one sample. The number of samples that the KO was present in is reported in the “Total Samples” column, and the number of samples that it was identified as being expressed by *C. difficile* is reported in the “Samples Associated with *C. difficile* Expression” column.

*Clostridioides difficile* was one of the taxa found to be significantly more critical to the expression of differential rRNA genes in CDI+ (Supplementary Table S7). It was also found to be a significantly more important contributor to the expression of three differential KOs related to spore formation in CDI+ (Supplementary Table S8). Likewise, *Enterocloster bolteae* contributed to the differential expression of two of those KOs (Supplementary Table S8). Similarly, though it was not associated with differential expression of those three spore formation KOs, *Ruminoccocus gnavus* expressed genes relating to spore formation as well. *Clostridioides difficile* also constituted a greater proportion of the expression of genes relating to biofilm formation in CDI+, specifically K03073 (*SecE*), K03075 (*SecG*), and K03666 (*hfq*; Supplementary Tables S9, S10). *Clostridioides difficile* and *R. gnavus* were also significantly more important to the expression of genes within the differential Flagellar Assembly (ko02040) in CDI+ (Supplementary Table S11). Additionally, *C. difficile* was identified as expressing the *tcdB* gene in three CDI+ samples (13, 19, and 5), albeit very few sequences were associated with both (1, 2, and 5 sequences respectively).

**Figure 6:**
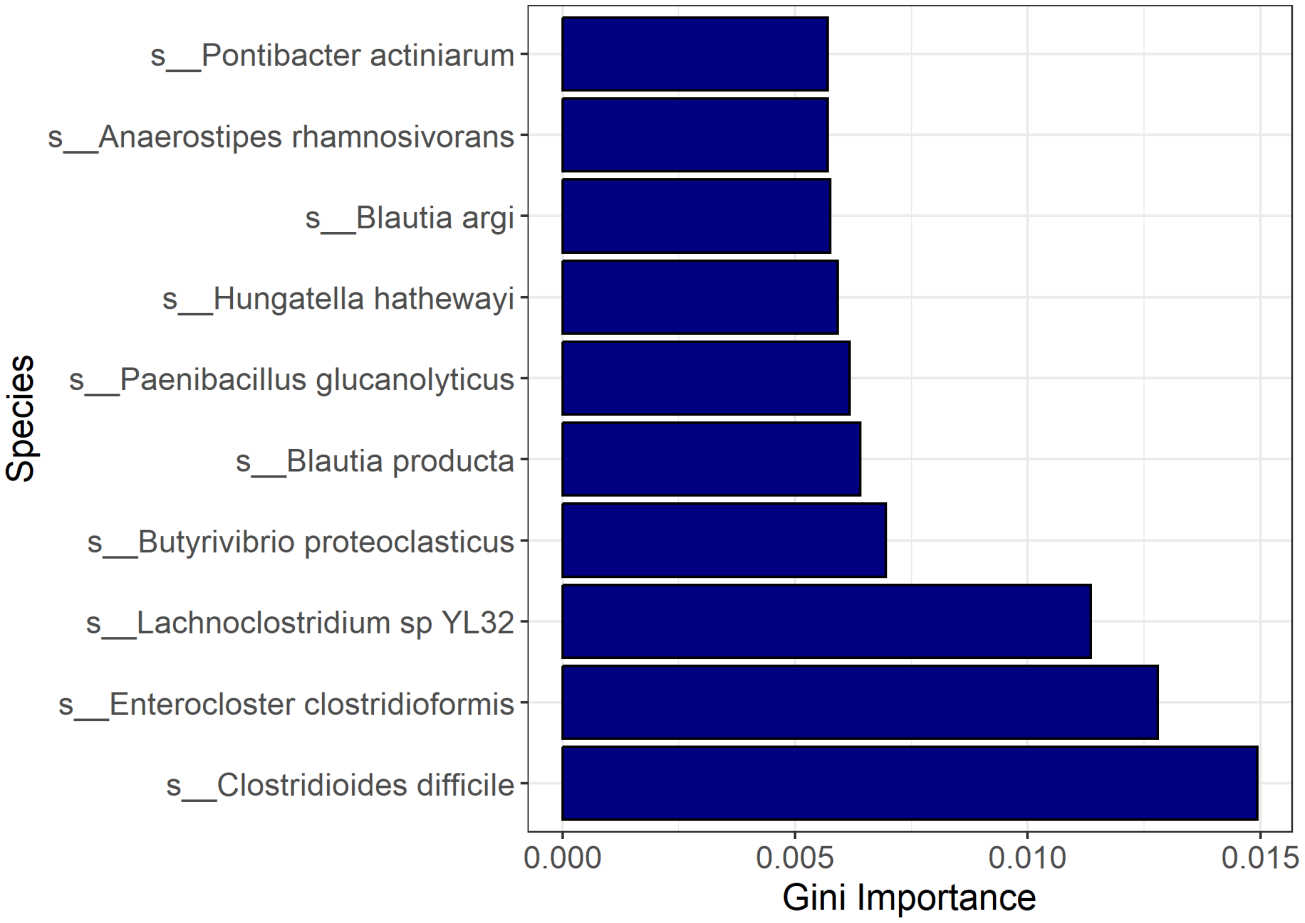
Bar plot of most important features for classification with random forest based on the full active microbial taxa dataset. Feature importance was measured by the decrease in impurity after splitting by the feature (Gini Importance). *Pontibacter actiniarum* and *Butyrivibrio proteoclasticus* were not identified as differential (LDA ≥ 2.0, Kruskal-Wallis *p* ≤ 0.05) by LEfSe.

Random forest modeling was used to help evaluate the consistency of features identified by LEfSe for differentiating samples based on CDI status, regardless of possible confounding factors. The performance of those models indicates that both microbial taxa and expressed genes identified by LEfSe differed consistently between CDI+ and CDI–. Both random forest models using differential features (genes or species) had average accuracies of 84.2% (Table 3). As expected, the differential species model excluding *C. difficile* performed worse, but it still had a 9% greater average accuracy than the random forest model that was generated using all species. Likewise, the genes model had a 7% increase in average accuracy between the dataset containing all genes and the dataset containing only genes identified as differentially expressed by LEfSe. Additionally, eight of the 10 best predictors for the full species dataset were also identified as being predictive of CDI status by LEfSe (Figure 6). The best predictors for the other models are shown in Supplementary Figures S5–S9.

**Table 3:** Random forest classification accuracy.

Model input	Average accuracy
All microbial species	69.5%
LEfSe identified differential species	84.2%
All microbial species without *Clostridioides difficile*	70.2%
LEfSe identified differential species without *C. difficile*	78.5%
All genes	77.2%
LEfSe identified differential genes	84.2%

Random forest modeling was performed using the scikit-learn package with Python.

### 3.4 Cross domain interactions

**Figure 7:**
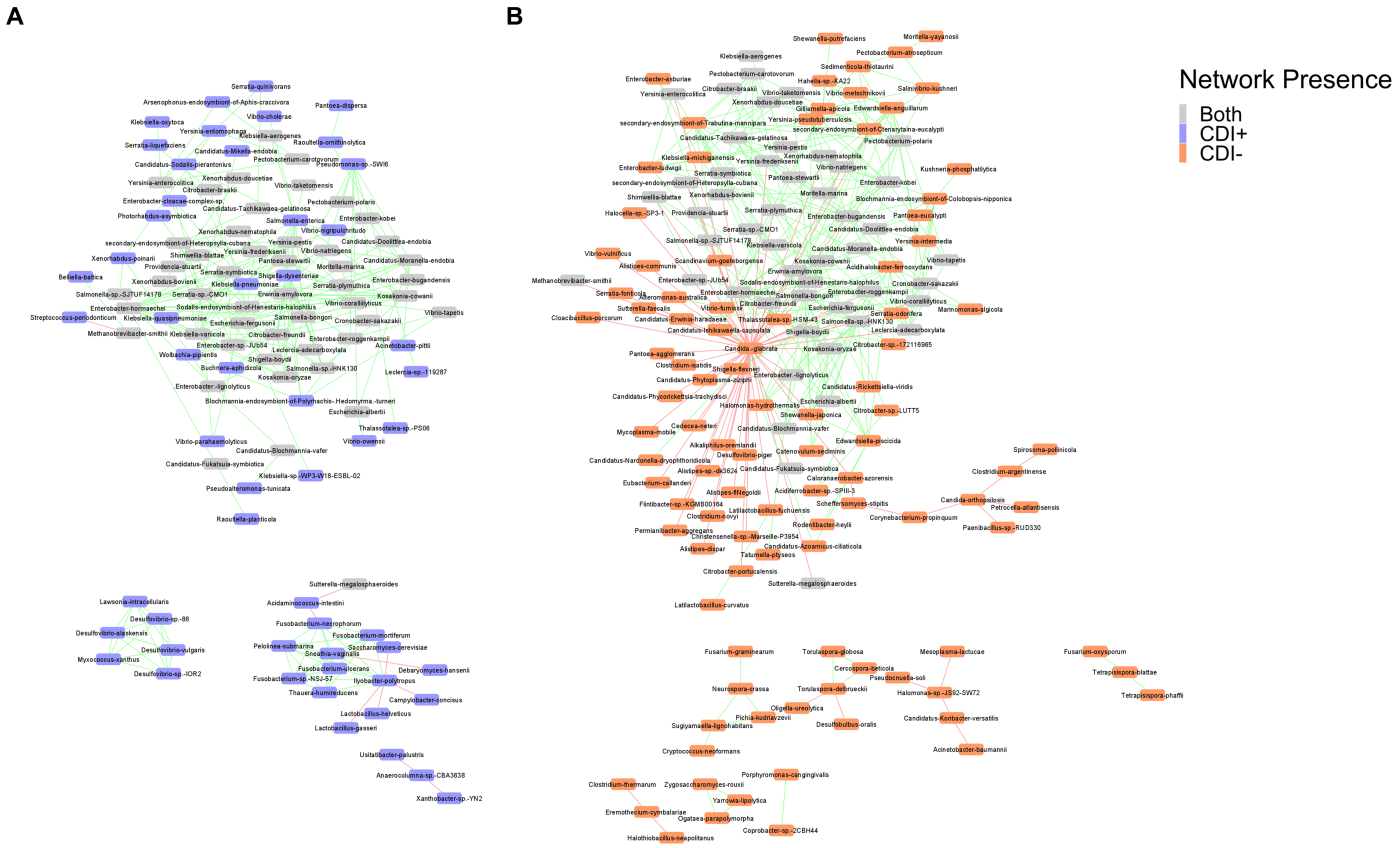
Co-occurrence networks for CDI+ **(A)** and CDI− **(B)**. Nodes are labeled by species name and colored by network presence. Edges showing positive correlations between the connected nodes are shown in green, and edges showing negative correlations are shown in red. Pairs of nodes that were only connected to each other were omitted from the final networks.

Co-occurrence network analysis helped us model potential transkingdom interactions between bacteria and fungi with respect to CDI status. The networks revealed negative relationships between fungi and bacterial taxa in both CDI− and CDI+ networks. In the CDI− network, 18 fungi were present, with *Nakaseomyces glabratus* (formerly *Candida glabrata*) having negative correlations with 57 taxa, 55 of which were bacteria (Figure 7). In contrast, *N. glabratus* was also entirely absent in the CDI+ network (Figure 7). Furthermore, the CDI+ network only had two fungi, *Saccharomyces cerevisiae* and *Debaryomyces hansenii*.

### 3.5 Predictive metabolic modeling

**Figure 8:**
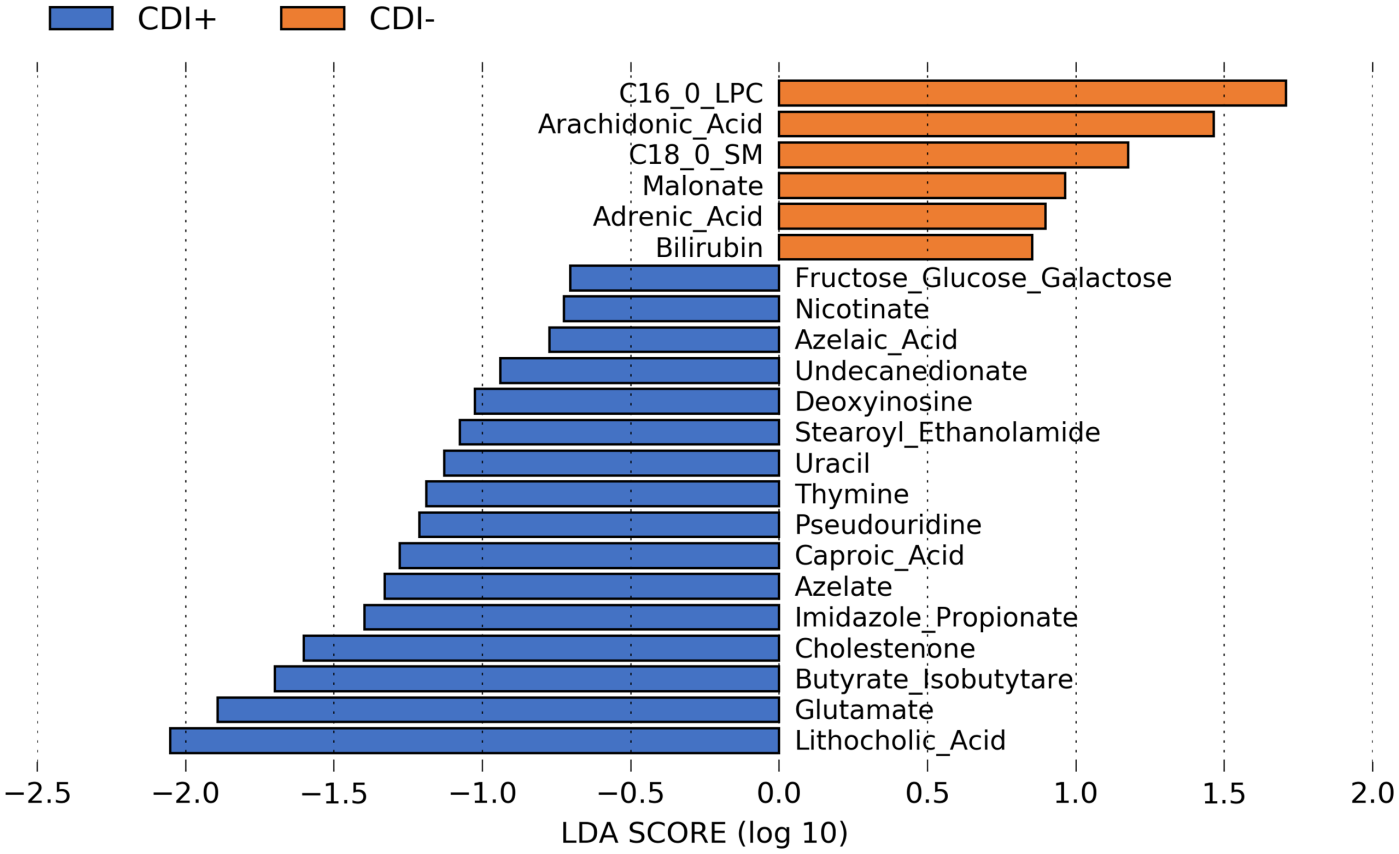
LEfSe bar plot showing differentially abundant predicted metabolites resulting from MelonnPan analysis (Kruskal-Wallis *p* ≤ 0.05) based on CDI status.

Several predicted metabolites (*n* = 16) were found to be significantly more abundant (Kruskal-Wallis, *p* ≤ 0.05; Supplementary Table S12) in CDI+ patients, including caproic acid (Figure 8). In addition, a secondary bile acid, lithocholic acid, was also predicted to be more abundant in CDI+. Fewer predicted metabolites (*n* = 6) were associated with CDI–.

## 4 Discussion

### 4.1 Microbiome differences

Although previous studies have noted significant differences in alpha diversity, those studies utilized 16S rRNA and ITS data to characterize microbial communities (Antharam et al., 2013; Lamendella et al., 2018; Zuo et al., 2018). To the best of our knowledge, this study is the first to examine possible alpha diversity differences based on CDI status using MT data. However, these results are aligned with another study that compared CDI− diarrheal communities to CDI+ communities that also did not observe significant differences between those groups (Antharam et al., 2013).

Still, similar to other CDI microbiome studies (Lamendella et al., 2018; Zuo et al., 2018; Stewart et al., 2019) overall community composition and gene expression differed significantly based on CDI status. The recapitulation of this finding is notable because these results indicate that both the active community members and the functions they express differ with respect to CDI status.

The species most strongly associated with CDI− samples are beneficial to human health. Specifically, both *Clostridium butyricum* and *Roseburia intestinalis* are butyrate producers (Hayashi et al., 2021; Nie et al., 2021). Similarly, the use of *Lacticaseibacillus rhamnosus* as a probiotic has been associated with increased butyrate production (Berni Canani et al., 2016; Lin et al., 2020; Carucci et al., 2022), and *Clostridium* and *Roseburia* genera are contained in a newly FDA-approved fecal microbiota capsule (SER-109) for use against recurrent CDI (Khanna et al., 2016). In the context of CDI, butyrate has been found to reduce inflammation and improve intestinal barrier function following *C. difficile* colonization (Fachi et al., 2019). *Roseburia intestinalis* was most abundant in the healthy cohort in a study examining the gut microbiome of children based on CDI and hospitalization (IC vs. non-IC) status (Mohandas et al., 2020). Additionally, multiple studies have found *C. butryicum* promotes resistance to *C. difficile* infection (Hagihara et al., 2021; Hayashi et al., 2021), and it has been investigated as a possible treatment for CDI (Oka et al., 2018; Lee et al., 2022). Likewise, studies have found evidence administering *Lactiplantibacillus plantarum* as a probiotic may help prevent CDI (Klarin et al., 2008; Kujawa-Szewieczek et al., 2015; Dudzicz et al., 2018). Therefore, the higher activity of *C. butryicum* and *L. plantarum* in CDI− samples could have helped protect those patients against acquiring *C. difficile*.

Multiple butyrate producers were also identified as more active in CDI+. Although previous research has shown that butyrate was significantly reduced in CDI cohorts compared to healthy controls (Pensinger et al., 2023), another study did not find a significant difference in butyrate producers (based on 16S rRNA analysis) in their diarrheal controls compared to CDI, while their healthy cohort had significantly more butyrate producers compared to their CDI and diarrheal control groups (Antharam et al., 2013). Therefore, since both CDI+ and CDI− groups in this study were comprised of diarrheal samples, differential gene expression attributed to butyrate producers in both is consistent with previous findings.

Interestingly, *Clostridium scindens* was also identified as being more active in CDI+. *Clostridium scindens* had previously been associated with resistance to CDI as a result of producing secondary bile acids (Buffie et al., 2015; Greathouse et al., 2015), but resistance may be contingent on the resulting concentration of deoxycholate (Dubois et al., 2019). Still, *C. scindens* has also been found to inhibit *C. difficile* growth independent of bile acid production (Aguirre et al., 2021). Instead, the authors of that study suggested resistance could be due to competition for nutrients with *C. difficile* (Aguirre et al., 2021). Therefore, though unexpected, if nutrient conditions were conducive to *C. difficile* growth in our CDI+ patients, increased activity of *C. scindens* could have also been favored.

Besides *Clostridioides difficile*, the other species most strongly associated with CDI+ are also known pathogens. *Enterocloster bolteae* has been previously noted to be an opportunistic pathogen and are also correlated with the expression of inflammatory genes (Pandit et al., 2021). Similarly, *Ruminococcus gnavus* has been found to produce a glucorhamnan that causes inflammation (Henke et al., 2019). Additionally, *Ruminoccocus gnavus* was previously associated with CDI in another study that found several operational taxonomic units (OTUs) belonging to this species were more abundant in their CDI IBD group compared to the non-CDI IBD group and the healthy subjects (Sokol et al., 2017). Notably, *Ruminococcus gnavus* is a mucin degrader, and a previous study found *C. difficile* grown in the presence of MUC2 with mucin-degrading taxa also present had increased expression of genes associated with flagella (Engevik et al., 2021).

The larger number of fungal taxa associated with CDI+ aligns with previous studies that found fungal taxa were more abundant in CDI patients (Sangster et al., 2016; Lamendella et al., 2018; Stewart et al., 2019). Therefore, this study provides further evidence that the mycobiome may have an important impact on the pathogenesis of CDI. For instance, *Encephalitozoon cuniculi*, a species of fungi enriched in CDI+ patients, is a known opportunistic pathogen (Moretto et al., 2015). Conversely, the use of *Saccharomyces boulardii,* which is a part of the family Saccharomycetaceae (more active in CDI− patients), as a probiotic seems to reduce CDI-associated diarrhea (Goldenberg et al., 2017).

The relatively large number of differential KEGG Orthologs associated with ribosomal subunits was likely due to the taxa expressing them being more transcriptionally active in CDI+ samples because of other reasons, rather than a driving reason for increased activity themselves. Higher expression of genes coding for ribosomes has previously been correlated with an increase in activity for the microbes expressing them, though not consistently (Blazewicz et al., 2013). Still, the relatively greater contribution of CDI+ differential taxa to these genes provides another line of evidence that they were more active in CDI+.

*Clostridioides difficile* is known to form spores during infection (Paredes-Sabja et al., 2014), which fits with the observation of it as a significantly more important contributor to the expression of three differential KOs related to spore formation in CDI+ in this study. Likewise, *Enterocloster bolteae* can form spores (Magdy Wasfy et al., 2023) and contributed to the differential expression of two of those KOs (Supplementary Table S8). Similarly, though *Ruminoccocus gnavus* had historically been considered to be non-spore forming, more recent work has demonstrated that it can form spores under certain conditions (Crost et al., 2023). Therefore, it is possible that members of all three taxa could form spores in response to antibiotics, which may help explain their presence in CDI+ samples, as antibiotics are often used to treat CDI.

Both *SecE* and *SecG* have previously been proposed to be involved with *C. difficile* biofilm (Poquet et al., 2018). *SecE* and *SecG* have also been associated with biofilm formation in *Staphylococcus aureus* (Resch et al., 2005) and *Bifidobacterium longum* (Zhang et al., 2023) respectively. Therefore, although we did not recapture our previous finding of differential biofilm pathways (Stewart et al., 2019), the observed overall increased expression of *hfq*, *SecE* and *SecG* and their expression by *C. difficile* in this study, as well as other genes relating to biofilm formation, provides further *in vivo* evidence that biofilm formation is involved in the pathogenesis of CDI. However, *hfq*, *SecE*, and *SecG* are also components of other pathways, and of the 21 KOs expressed by *C. difficile* that are associated with at least one biofilm formation pathway, only two are associated solely with biofilm formation (Supplementary Table S10). Therefore, future work is needed to confirm *in vivo* biofilm formation. Still, biofilm formation, if occurring, could help protect *C. difficile* from antibiotics and consequently, contribute to CDI’s high reoccurrence rate.

Two of the four pathways more expressed in CDI+ [Ethylbenzene Degradation (ko00642) and Flagellar Assembly (ko02040)] were also identified as being differentially expressed in our prior study (Stewart et al., 2019). Notably, the production of flagella has been associated with pathogen colonization (Rossez et al., 2015), and *C. difficile*, *E. bolteae*, and *Parabacteroides distasonis* were significantly more important contributors to genes within that pathway in CDI+ (Supplementary Table S11). Like *E. bolteae*, *P. distasonis* is also an opportunistic pathogen (Ezeji et al., 2021). Therefore, the enrichment of the flagellar assembly pathway here was driven by the increased activity of potentially pathogenic bacteria.

Correlations between bacteria and fungi within CDI+ samples have been observed in other studies (Lamendella et al., 2018; Stewart et al., 2019). However, Lamendella et al., 2018 found no correlations between fungi and bacteria in CDI− (Lamendella et al., 2018), and Stewart et al., 2019 had only one unidentified fungi with a significant correlation to an unidentified *Bacteroides* OTU (Stewart et al., 2019). Networks in both were based on ITS data, so the use of MT data here instead is a likely contributor to the differences in the prevalence of fungi in the CDI− network. Still, despite evidence of fungi’s importance to CDI pathogenesis, co-occurrence analysis itself did not identify a significant correlation between fungal species and *C. difficile* in this study or in either of those previous studies (Lamendella et al., 2018; Stewart et al., 2019).

Caproic acid was previously detected in 41% of CDI+ fecal samples according to a gas chromatography study used to predict *C. difficile* (Levett, 1984). In contrast, lithocholic acid, also associated with CDI+ in this study, has previously been found to inhibit the growth of *C. difficile* (Kang et al., 2019). However, it is possible that lithocholic acid could have been more abundant in our CDI+ samples but still below a concentration that would have been lethal to *C. difficile*, and if so, then it is also possible that lithocholic acid could have induced biofilm formation, as was the case with deoxycholate in a previous study (Dubois et al., 2019). This possibility is supported by our observance of *C. difficile* expressing multiple genes related to biofilm formation.

Still, it should be noted that tools like MelonnPan are predictive metabolic modeling tools, and it is unknown whether the lithocholic acid metabolite was truly more abundant in our CDI+ samples. Consequently, directly measuring metabolites of interest like lithocholic acid and caproic acid, in conjunction with microbiome analysis, would be valuable to further understanding how the abundance of metabolites previously associated with CDI relates to microbiome composition and function.

### 4.2 Limitations

Unfortunately, no information about antibiotic usage or clinical history was reported for the individuals used in this study. Previous work has shown that antibiotic usage in conjunction with CDI has a large impact on the host’s overall microbial community (Lamendella et al., 2018). Specifically, in that study, the fidaxomicin and metronidazole groups had significant differences in alpha and beta diversity between CDI+ and CDI−, while the vancomycin group did not (Lamendella et al., 2018). Additionally, different taxa were detected as being significantly differential between CDI+/− depending on the antibiotic class (Lamendella et al., 2018). Still, another study did not find a significant difference in alpha diversity within their CDI+ and CDI− groups when split by antibiotics exposure (Antharam et al., 2013). However, it is possible that a differential usage of antibiotics could have impacted our results. Consequently, we limited much of our discussion to comparing the results obtained herein to previous CDI studies.

The failure to consistently capture sequences associated with both *C. difficile* and the gene used for diagnosis (*tcdB*) could also indicate our sequencing depth was not sufficient to fully capture all expressed genes in our samples. Additionally, due to the cost associated with MT, our sample size was smaller than some previous studies. Though, *post hoc* power analysis using SparseDossa2 showed we had reasonable (>80%) power to detect changes corresponding to at least 7.5 log2 fold change in the active species dataset and 4.5 log2 fold in the expressed genes dataset with LEfSe.

### 4.3 Conclusions and future directions

Despite antibiotic usage being a potentially large confounding variable, this work has contributed great value in characterizing active microbes and their associated functions in the context of CDI. To the best of our knowledge, MT analysis had only been applied to human samples associated with this disease state in a previous study by our group, in which it was used to identify differential genes and pathways (Stewart et al., 2019). Therefore, this study represents one of the first applications of MT to characterize differences in overall community diversity between CDI+ and CDI− cohorts. Although alpha diversity did not differ significantly, beta diversity for active species and expressed genes differentiated CDI+ and CDI− cohorts.

Subsequent differential feature analysis identified the specific genes and taxa driving those overall differences, revealing significant increases in the activity of several beneficial bacteria in CDI− and increased activity of taxa associated with inflammation in CDI+. Although the previous finding of differential biofilm formation pathways (Stewart et al., 2019) was not recaptured, several specific genes associated with biofilm formation were identified as differentially expressed in CDI+, and *C. difficile* was identified as a contributor to them, serving as additional evidence for the role of biofilm formation in CDI pathogenesis. Biofilm formation during infection would help explain CDI’s high recurrence rate, and if that is the case, treatments designed to target biofilms specifically could be effective for preventing reoccurrence.

Additionally, multiple fungal taxa were identified as being more transcriptionally active in CDI patients, which provides additional support for the potential importance of the mycobiome to CDI. Notably, *E. cuniculi* can cause inflammation, and increased inflammation due to it or other fungi could be one mechanism by which severe CDI could occur irrespective of bacterial toxin production. However, coverage for both *E. cuniculi* and fungi overall in this study tended to be low. Determining if specific fungal taxa are important for preventing or enabling CDI could facilitate the use of additional treatment methods. The low proportion of CDI+ samples that had *tcdB* identified within them suggests that greater sequencing coverage would also be valuable for characterizing the functional capabilities of these microbial communities. Therefore, future work should include deeper metatranscriptomics sequencing in order to increase coverage of the entire microbial community.

Increasing fungal sequences specifically, either through overall increased sequencing depth or targeting sequencing, would also aid better understanding if fungal activity contributes to the pathogenesis of CDI. Likewise, the development of methods to increase the proportion of fungal nucleic acids recovered would similarly help facilitate investigations of fungi’s role in CDI. Additionally, future iterations of the CDI status model could theoretically be trained to differentiate non-*C. difficile* diarrhea with colonization from clinically-relevant *C. difficile* infection, as a means to reduce unnecessary anti-*C. difficile* antibiotic use (Polage et al., 2015). Metabolomics data would also be invaluable for further exploring the role that previously identified metabolites of interest play in CDI.

## Data availability statement

Raw sequencing data are available from NCBI’s Short Read Archive under BioProject ID PRJNA1035947.

## Ethics statement

The studies involving humans were approved by Institutional Review Board at the University of Virginia. The studies were conducted in accordance with the local legislation and institutional requirements. The ethics committee/institutional review board waived the requirement of written informed consent for participation from the participants or the participants’ legal guardians/next of kin because samples were de-identified remnants.

## Author contributions

JC: Formal analysis, Visualization, Writing – original draft, Writing – review & editing. JL: Investigation, Writing – review & editing. JW: Resources, Supervision, Writing – review & editing. PK: Formal analysis, Writing – review & editing. MK: Formal analysis, Writing – original draft, Writing – review & editing. CB: Formal analysis, Writing – original draft, Writing – review & editing. CK: Conceptualization, Writing – review & editing. GM: Conceptualization, Writing – review & editing. DS: Conceptualization, Writing – original draft, Writing – review & editing. RL: Conceptualization, Supervision, Writing – original draft, Writing – review & editing.
