# Supplementary material for: Clostridioides difficile infection is associated with differences in transcriptionally active microbial communities
Source: Front Microbiol. 2024 Apr 12;15:1398018. doi: 10.3389/fmicb.2024.1398018 (PMC11045941; doi:10.3389/fmicb.2024.1398018)
Supplement: Supplementary file 2 [file Data_Sheet_1.docx]

*Clostridioides difficile* Infection is Associated with Differences in Transcriptionally Active Microbial Communities

Jeremy R. Chen See^1^, Jillian Leister^1^, Justin R. Wright^1,2^, Peter I Kruse^1^, Mohini V. Khedekar^1^, Catharine E. Besch^1^, Carol A. Kumamoto^3^, Gregory R. Madden^4^, David B. Stewart^5^, Regina Lamendella^1*^

^1^Juniata College, Huntingdon, PA

^2^Wright Labs LLC, Huntingdon, PA

^3^Tufts University, Boston, MA

^4^University of Virginia School of Medicine, Charlottesville, VA

^5^Southern Illinois University School of Medicine, Springfield, IL

*** Correspondence:**Dr. Regina Lamendella
lamendella@juniata.edu

Keywords: *Clostridioides difficile*, Metatranscriptomics, Fungi, Human Microbiome, Mycobiome

**Supplemental Figures**


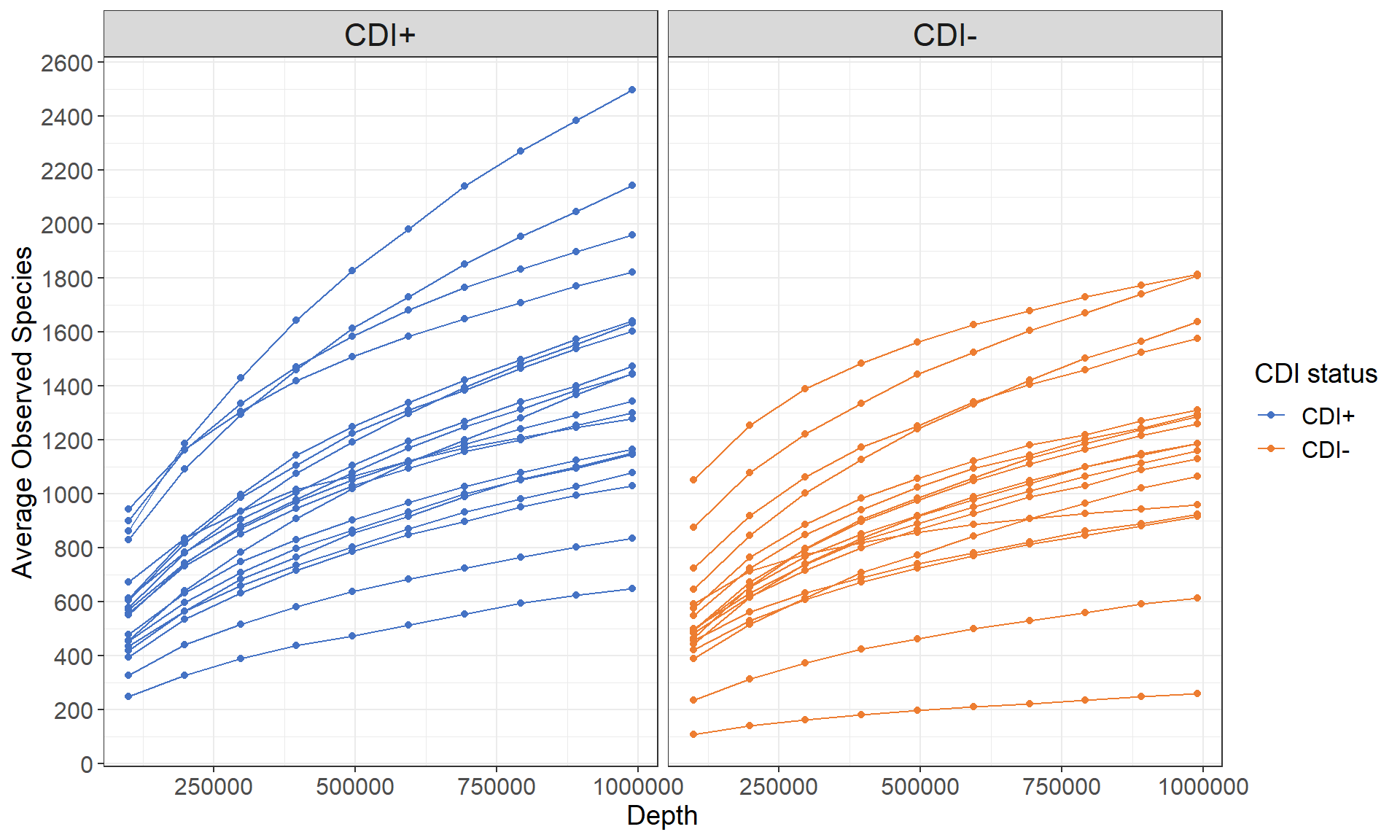


**Fig. S1**. Rarefaction curves based on the observed features alpha diversity metric for the active microbial species dataset. The average alpha diversity based on that metric is shown for each sample at every depth. Sample 68 had 117,641 sequences classified to the species level. Consequently, it was omitted from alpha diversity analyses for the active microbial species dataset to allow a rarefaction depth of 990,000 to be used.


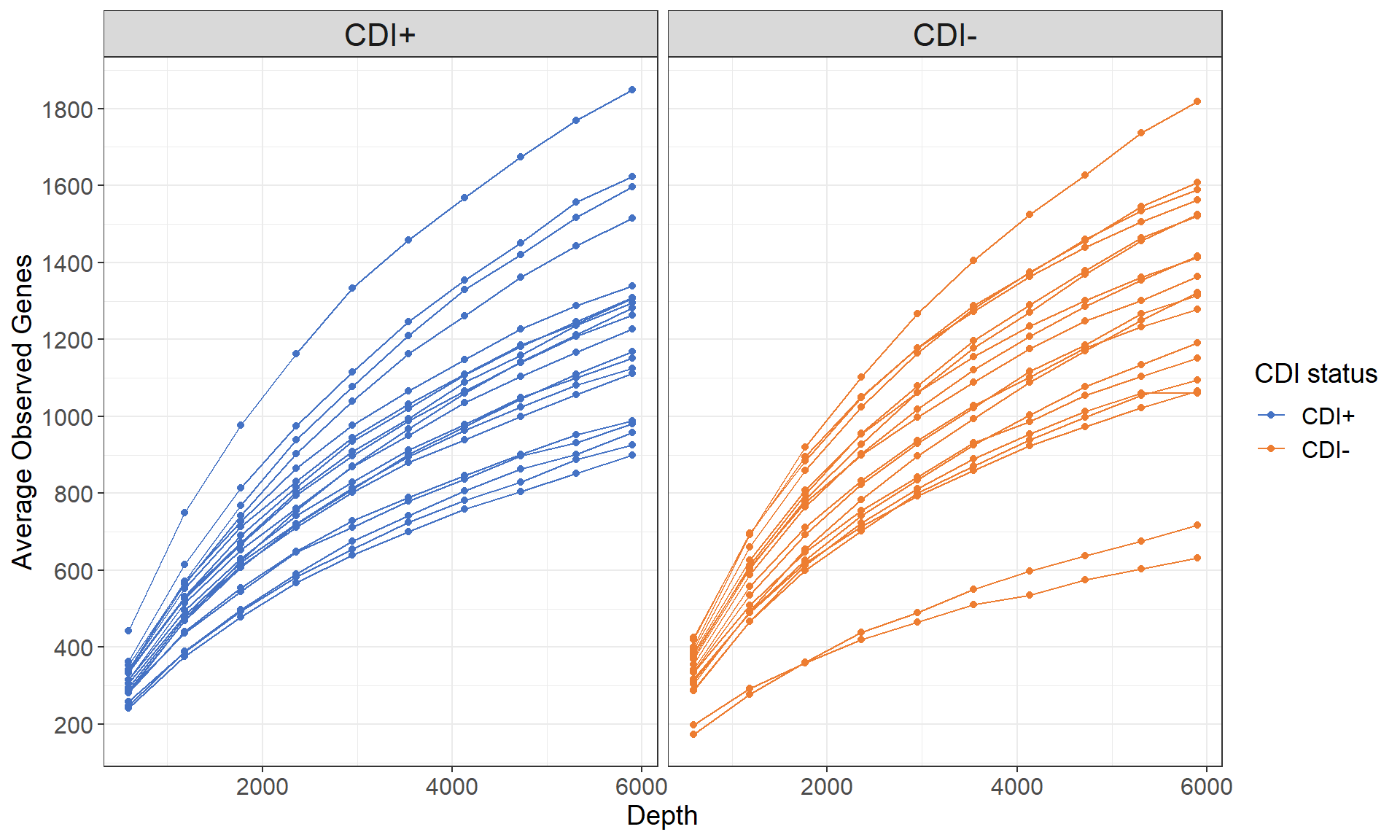


**Fig. S2**. Rarefaction curves based on the observed features alpha diversity metric for the expressed genes dataset. The average alpha diversity based on that metric is shown for each sample at every depth.


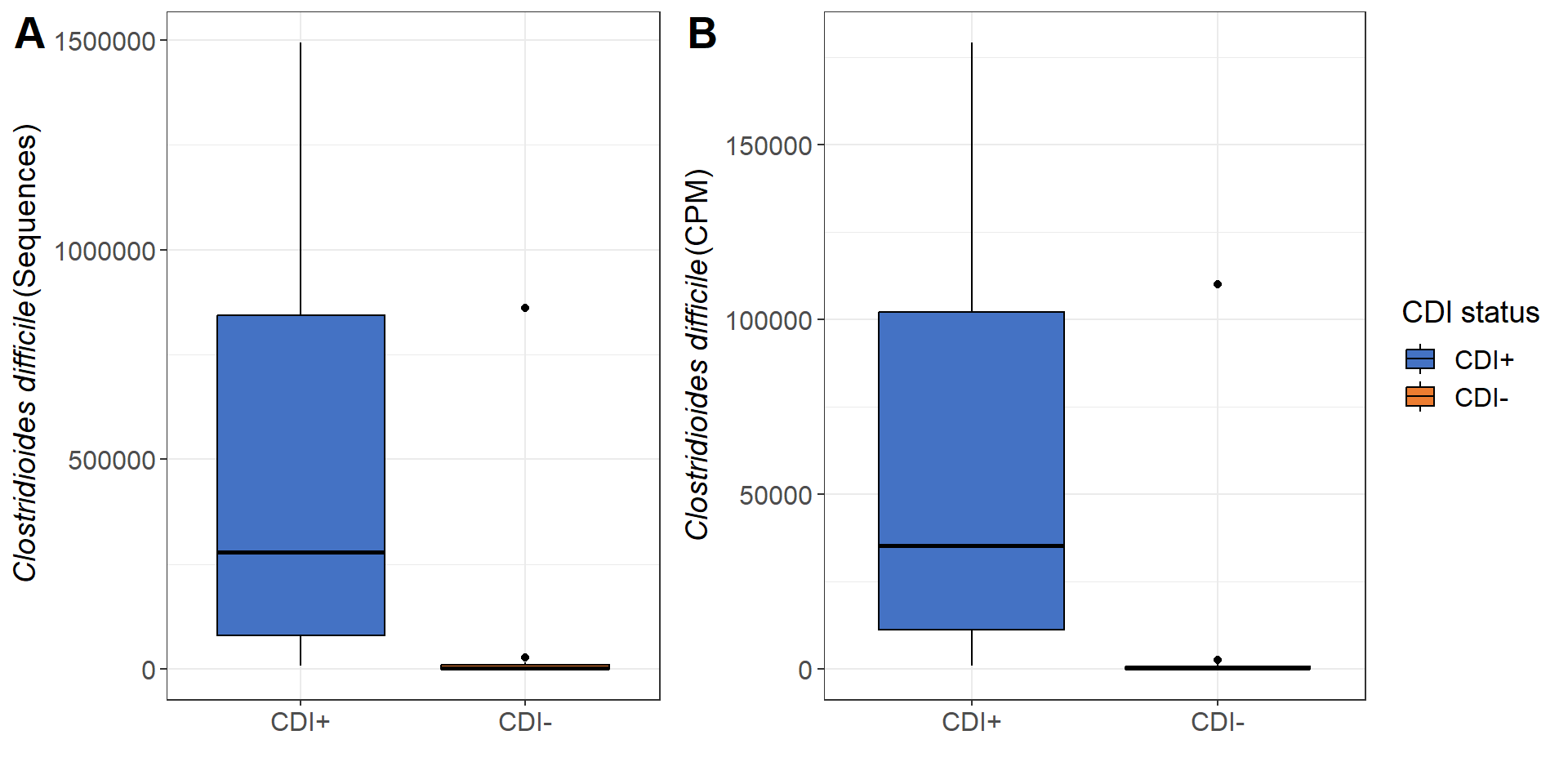


**Fig. S3**. Boxplot of *Clostridioides difficile* values in CDI+ and CDI- samples. (A) Number of raw sequences classified as *C. difficile*. (B) CPM-normalized *C. difficile* values.


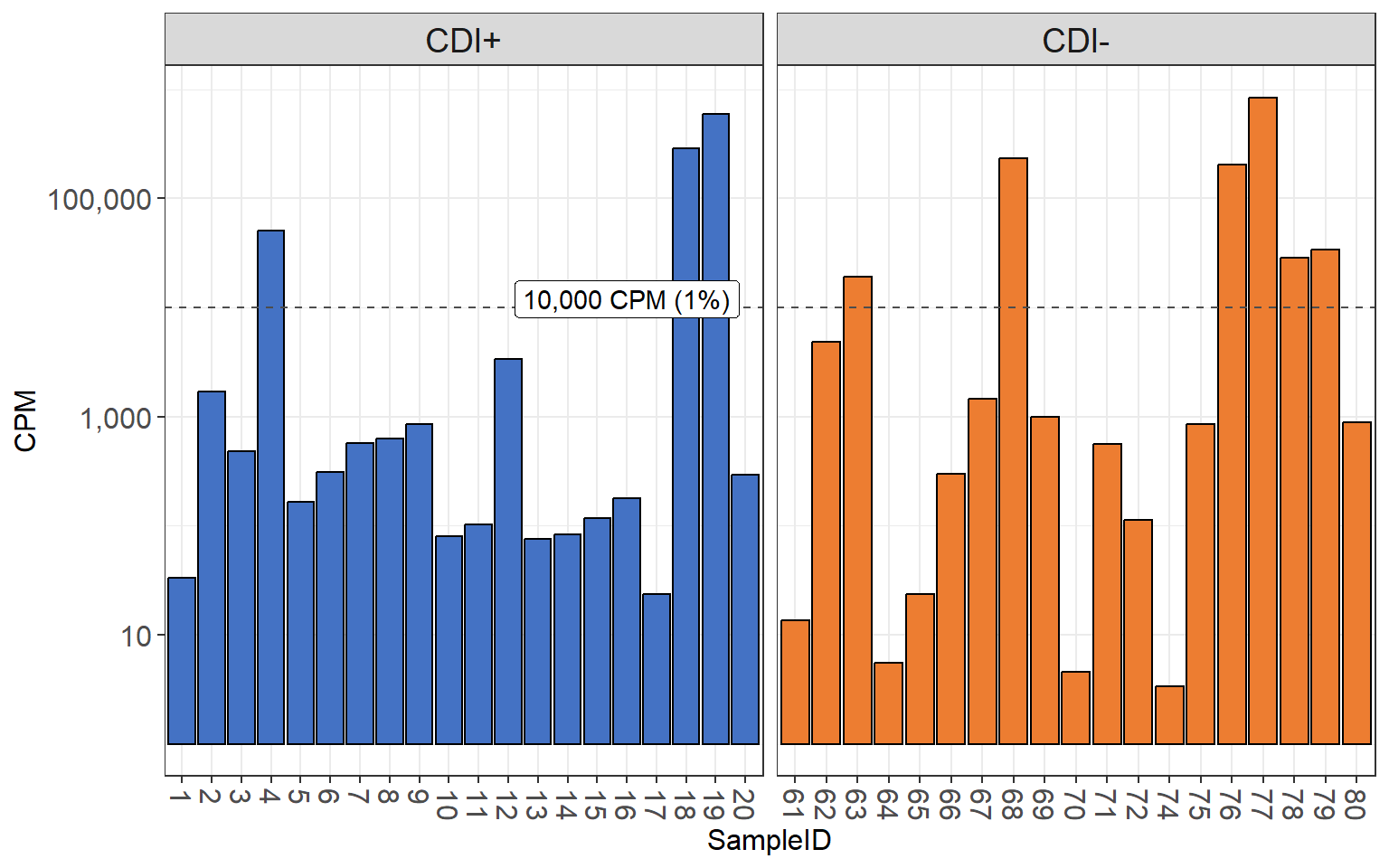


**Fig. S4**. Bar plot of fungal sequences. CPM values based on the number of fungi sequences are shown. Fungal sequences composed less than 1% of the Kraken2-annotated sequences in most samples.


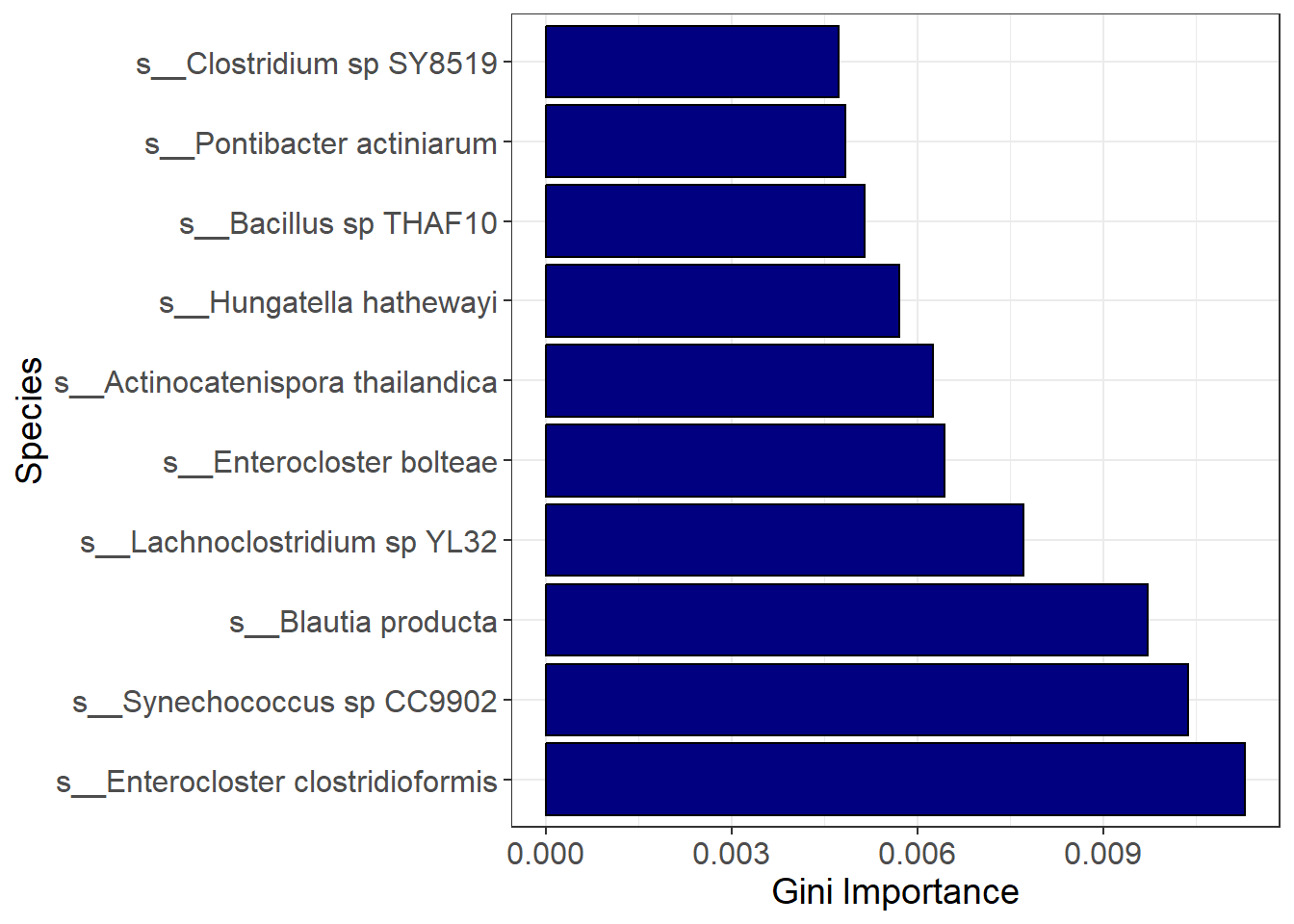


**Fig. S5**. Bar plot of the most important species for the random forest model based on the microbial taxa dataset with *Clostridioides difficile* excluded. Feature importance was measured by the decrease in impurity after splitting by the feature (Gini Importance).


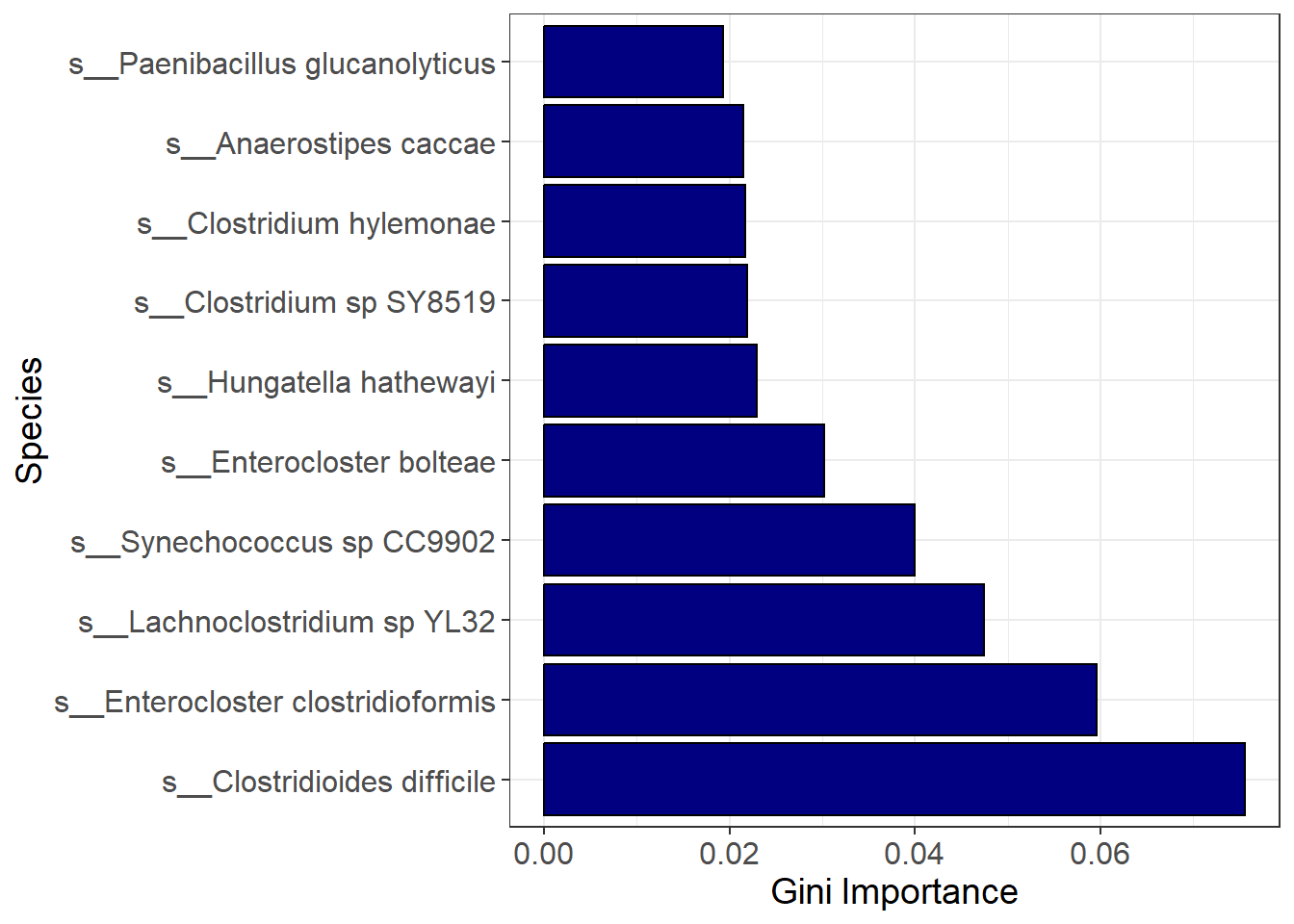


**Fig. S6**. Bar plot of the most important species for the random forest model based on the differential species identified within the microbial taxa dataset. Feature importance was measured by the decrease in impurity after splitting by the feature (Gini Importance).


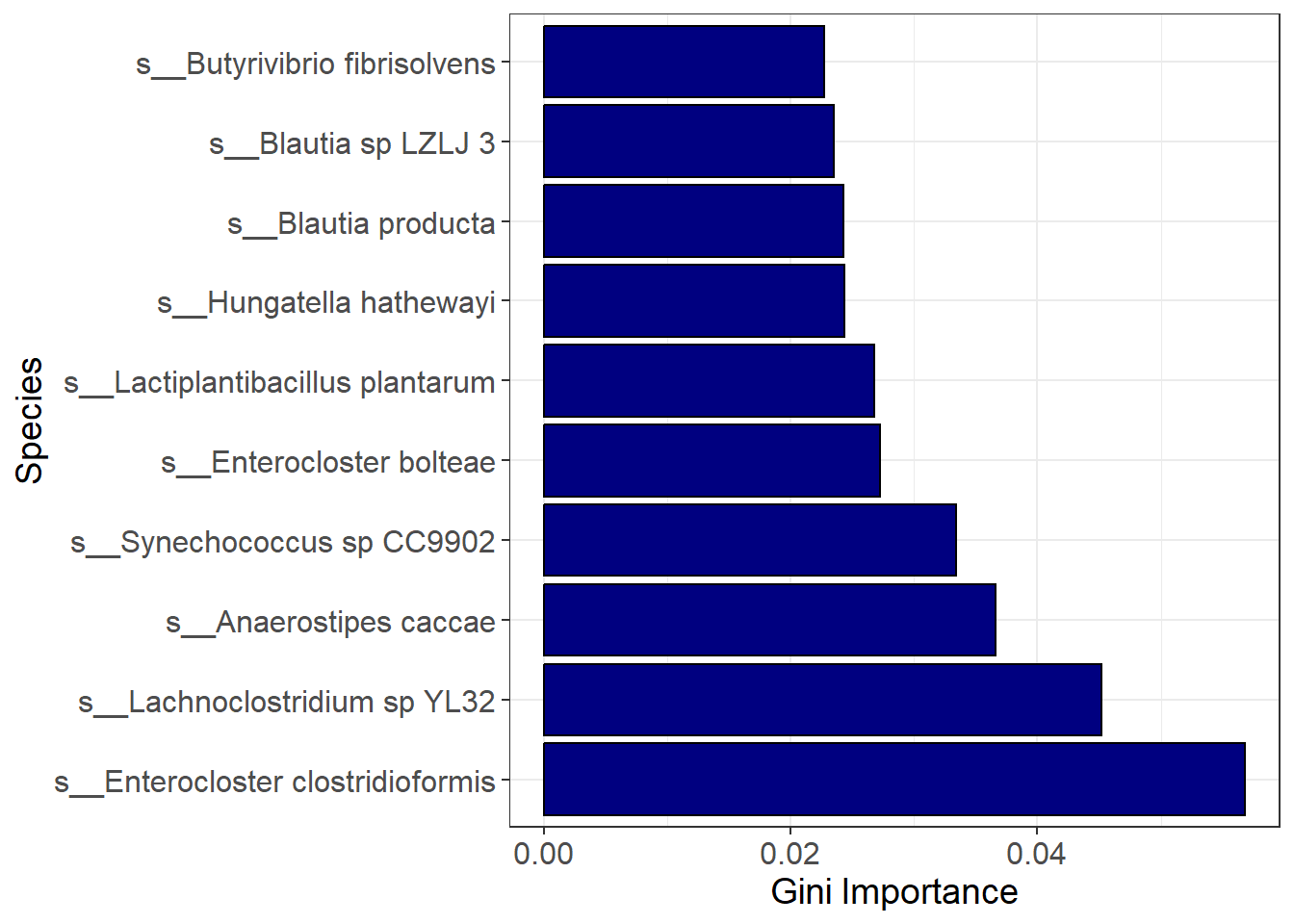


**Fig. S7**. Bar plot of the most important species for the random forest model based on the differential species identified within the microbial taxa dataset with *Clostridioides difficile* excluded. Feature importance was measured by the decrease in impurity after splitting by the feature (Gini Importance).


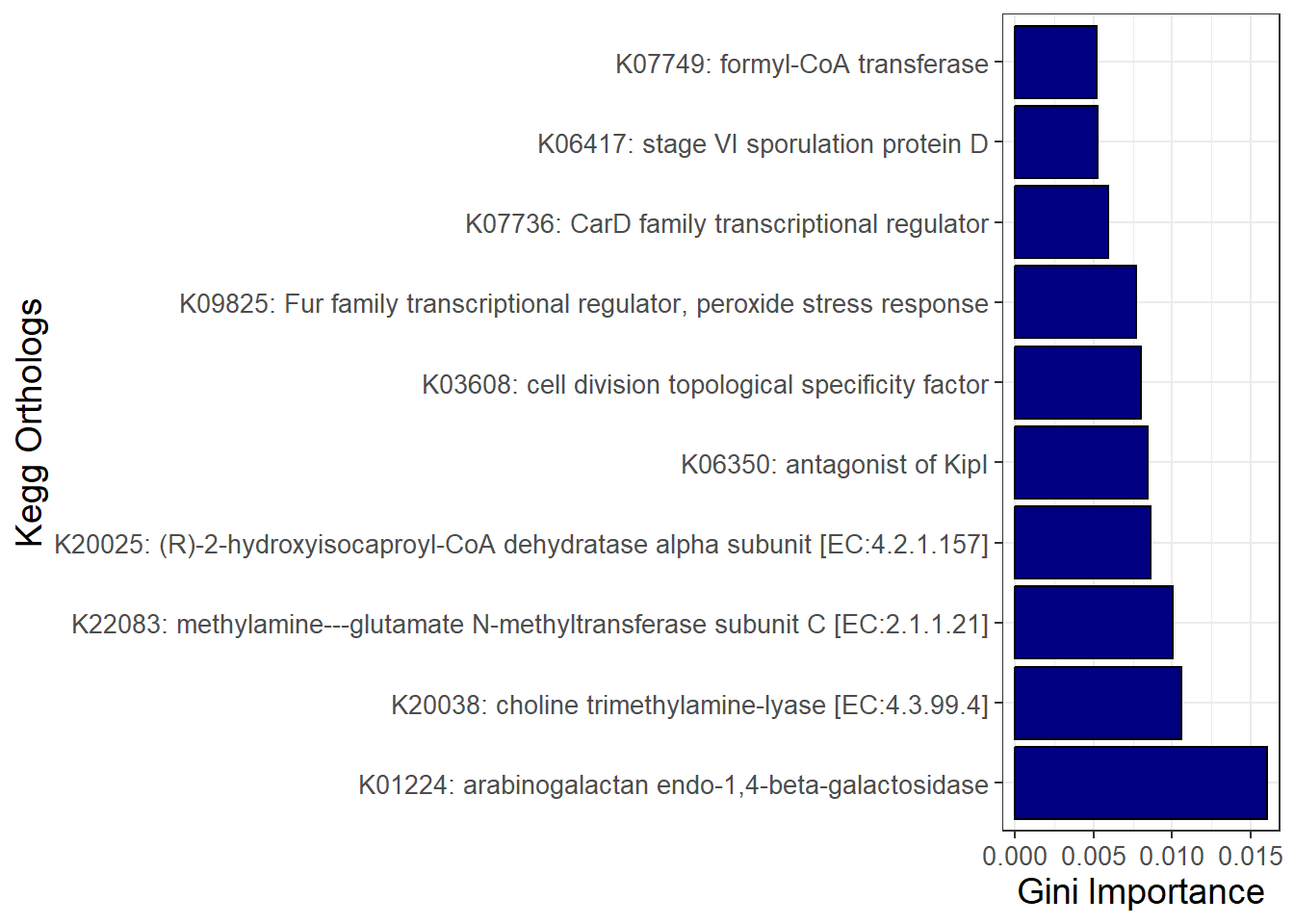


**Fig. S8**. Bar plot of the most important KEGG Orthologs for the random forest model based on the expressed genes dataset. Feature importance was measured by the decrease in impurity after splitting by the feature (Gini Importance).


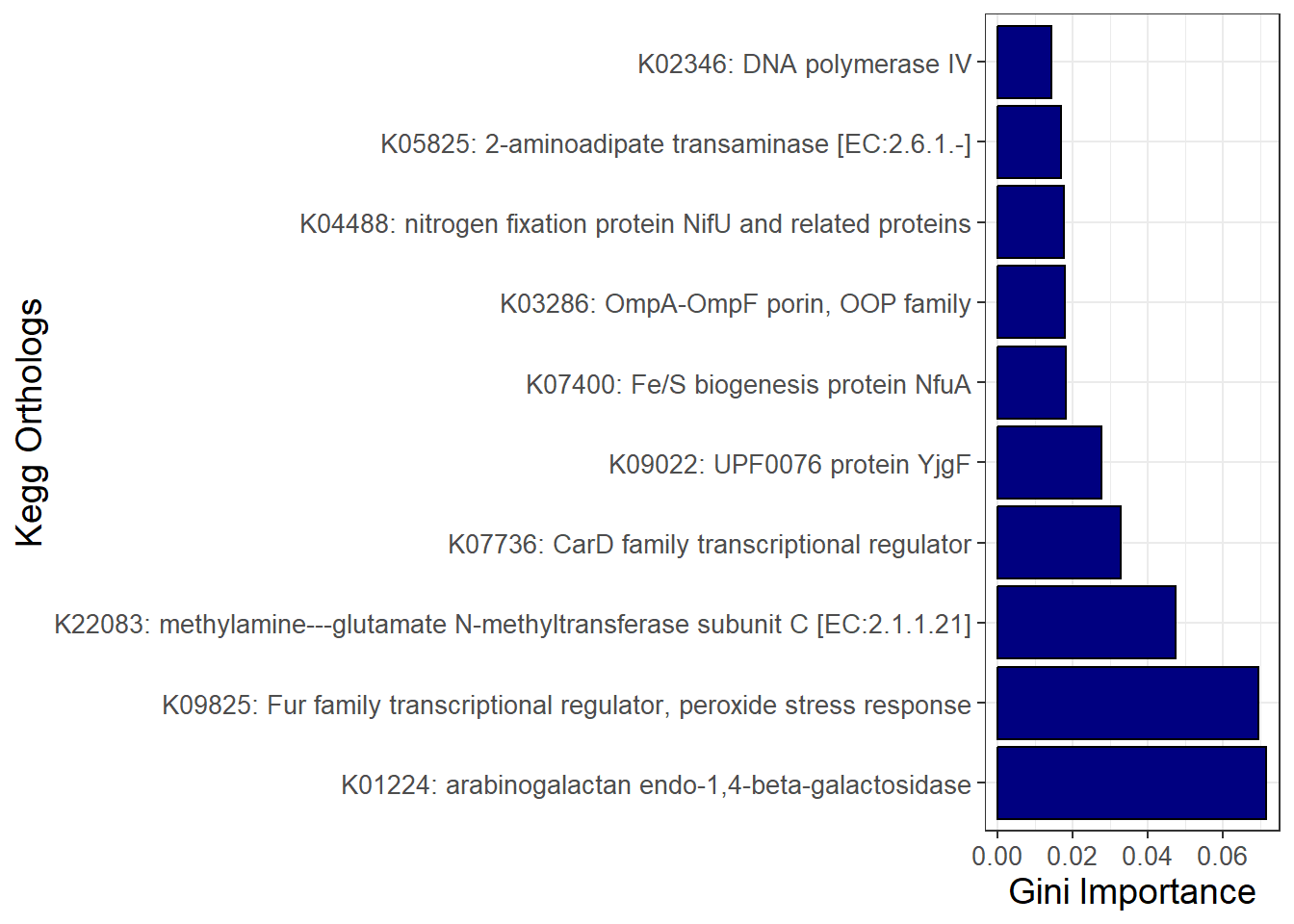


**Fig. S9**. Bar plot of the most important KEGG Orthologs for the random forest model based on the differential KEGG Orthologs identified within the expressed genes dataset. Feature importance was measured by the decrease in impurity after splitting by the feature (Gini Importance).
